# Design and Analysis of Health Products and Services: An Example at a Specialized COPD Unit

**DOI:** 10.2174/1874306400802010007

**Published:** 2008-02-20

**Authors:** Christian Domingo, Vicente Ortún Rubio

**Affiliations:** 1 Servei de Pneumologia Corporació Parc Taulí-Institut Universitari Parc Taulí-FPT Departament de Medicina-Universitat Autònoma de Bellaterra (UAB), (Barcelona) Àrea d’Anatomia i Fisologia, Facultat de Ciències de la Salut, Universitat Internacional de Catalunya (UIC), Barcelona, Spain; 2 Departament d’Economia i Empresa Centre de Recerca en Economia i Salut Universitat Pompeu Fabra, Barcelona, Spain

## Abstract

Health care demands have increased dramatically in recent decades. With the introduction of major changes in the management of health problems, health care costs have spiralled. Today, in the interests of cost control, medicine is geared towards outpatient care whenever possible.

In this process, the medical community has been obliged to adapt its traditional criteria to the dictates of national economies. Today the criteria for the organization and evaluation of the health services are based on the concepts of efficacy, effectiveness and efficiency. This has led to the emergence of a new discipline for the design and evaluation of medical service production, known as servuction, an amalgam of “service” and “production”. The organigram of a new health product should include the problems the program faces and the steps proposed to overcome these problems.

The concept of evaluation can be divided into two categories: administrative evaluation, and evaluative research. Avedis Donabedian was one of the founders of evaluative research, based on an easy-to-remember triad: structure-process-results. In the final evaluation of a new health care model, the innovations it provides must be considered.

In this article we describe the stages involved in the design of a new health product and correlate them with the types of evaluation that should be applied at each point in the process. Our discussion addresses general aspects of servuction, but also focuses on the design of a particular service, created to care for patients with severe COPD.

## INTRODUCTION

Health care organization has evolved considerably in the last 20 years, on the one hand in order to respond to the growing needs and demands of the population and on the other due to the pressure to control expenditure in an area that devours financial resources. In countries with state health planning systems, the rationalization of the process aims to limit health expenditure and at the same time to channel it towards the areas most at need. This has led to controversy between doctors and health managers (on occasion doctors who no longer practise, or doctors who may never in fact have practised at all).

In 1920, the Dawson report highlighted the importance of using epidemiological criteria in the design of the health services and the allocation of resources. The report also established for the first time the concept of different levels of health care and proposed the creation of primary health care centres [1]. This radical vision was largely ignored in the literature and it was not until 1978 that the World Health Organization (WHO) acknowledged the importance of primary care and defined accessibility, understanding, coordination and continuity as its key features. Around the same time, Wennberg [2] found that the costs of hospital treatment were lower than those of outpatient care, identifying the need for an important qualitative change in the focus of a publicly-funded health system. In the last century we have moved from a model in which health service consumption was predominantly hospital-based towards one in which expenditure is increasingly channelled towards the primary care level [3]. This process has been slow but remorseless, and physicians have often been unaware of it.

The fact is that, whenever possible, medicine today is geared towards outpatient care, in order to increase the comfort of patients and families while attempting to keep expenditure down. The onus is gradually shifting onto primary care. This process cannot occur *ad hoc*, as has classically been the case in medicine (for instance, the development of a service, structure or practice to respond to specific needs as they arise) since it requires a high degree of interrelation between different care levels. A careful prior design is essential to ensure efficiency. This is the job of health planning and management and is a process in which doctors have an important part to play.

**Fig. (1).**
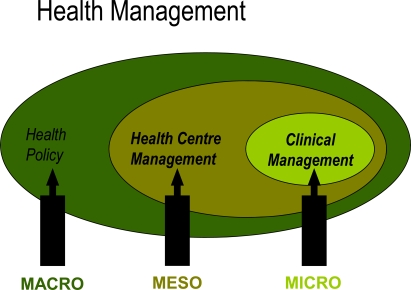


In Spain, a country with 40 million inhabitants, total health spending accounted for 8.2% of GDP in 2005, below the average of 9.0% in OECD countries. Health spending as a share of GDP is highest in the United States (which spent 15.3% of its GDP on health in 2005), followed by Switzerland (11.6%), France (11.1%) and Germany (10.7%). Spain also ranks below the OECD average in terms of health spending per capita, with spending of 2255 USD in 2005 (adjusted for purchasing power parity), compared with an OECD average of 2759 USD. Health management has three levels: macro, meso and micromanagement (Fig. **1**) [4]. In the context of micro health management or clinical management, doctors assign 70% of this total to their treatment decisions [5,6]. This means that clinical management has tremendous repercussions for the consumption of economic resources. The central problem facing the health services is to provide health professionals with the information they need to take cost-effective decisions and to encourage them to become involved in management, or clinical management, as it is now termed.

## CRITERIA FOR ORGANIZATION AND DESIGN

### Premises:

The current criteria for the organization and evaluation of the health services are based on the concepts of efficacy, effectiveness and efficiency. Efficacy measures the probability that an individual will benefit from a medical technology in ideal conditions. Broadly speaking, efficacy is useful to evaluate novel technologies and treatments. The tool used to determine it is the clinical trial. Like efficacy, effectiveness measures the probability that an individual will benefit from a medical technology, but this time in real, not ideal, conditions. This means that it will vary according to the organizational context and the society in question. The design most frequently used to determine effectiveness is the observational study. The concept of quality is linked to efficacy and effectiveness: the smaller the difference between them, the higher the quality of the service. Finally, when we introduce the concept of economic evaluation of a service, we speak of efficiency. Some time ago, in 1974, Sir Archibald Cochrane [7] stated that while efficacy had not been widely studied, efficiency had received even less attention.

### Organization:

A vital part of any assessment of health services (either new services, or those already in operation) is economic evaluation [8]. In the launch of any new service or product known today as servuction (an amalgam of the two key words, *ser*vice and *production)* it is essential to have a clear conceptualization of the model and of its utility, of the services the new product aims to replace and at which levels, and to be fully aware that the new service is likely to entail a supplementary cost that must be budgeted for.

## LEVELS AND TYPES OF EVALUATION IN THE CREATION OF A NEW HEALTH PRODUCT

Evaluating involves issuing a judgement on a resource, activity or outcome, by applying a series of pre-established criteria or standards. Criteria are indicators or variables used to measure how far a) the objectives are fulfilled, b) the program fits the objectives and c) the resources used are sufficient. Standards are the level of reference of a criterion which allows an evaluation to be carried out. Evaluation is an ongoing process designed above all to correct and improve aspects of the program in order to increase its effectiveness, suitability and efficiency. Evaluation is a key element in planning, design, management and decision-making, providing valuable information to allow modification of the program where necessary and to determine the program’s impact and outcomes.

The concept of evaluation can be divided into two categories. The first is administrative evaluation, which focuses on a particular aspect of the program, which aims to aid the decision-making process relating to specific resources or activities. Applying previously defined criteria and standards, administrative evaluation fine-tunes components of the program, checking or improving their functioning. The second is evaluative research which applies scientific methods to establish how, and how far, the interventions produce the desired effects. Its aims are to identify the effect of the programs or interventions, establish the validity of the standards and explain why a program succeeds or fails.

**Fig. (2).**
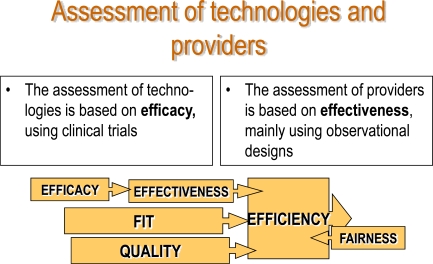


Evaluation can be applied both to technologies and to providers (see Fig. **2**). The ultimate aim is to promote efficacious interventions and to conduct them effectively and accurately in the right populations, seeking to achieve the greatest well-being possible while upholding the principle of fairness at all times. One problem with the evaluation of outcomes is that they do not depend exclusively on the effectiveness and efficiency of the technology or the provider, but also on the severity of patient’s condition. This means that in comparisons of several studies – a meta-analysis, for example – the prerequisites needed for a correct evaluation are not always met.

**Fig. (3).**
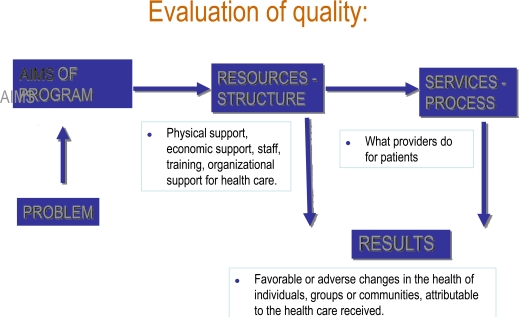


**Fig. (4).**
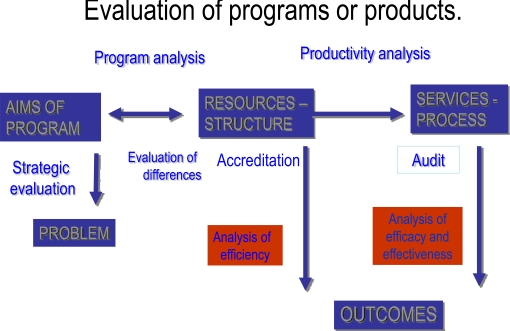


Avedis Donabedian was one of the founders of evaluative research, based on an easy-to-remember triad: structure-process-results (Fig. **3**). The organigram of a servuction should also include the problems the program faces and the steps proposed to overcome these problems. Once the organigram is complete, the quality of each of the steps and components must be evaluated. Fig. **4** presents these steps graphically:

- The first two steps refer to the planning and production of a servuction:
  **Strategic evaluation, **which establishes whether the program is indeed the best suited to solving the problem identified. Relates aims with problems.
  **Program analysis,** which establishes whether the program is correctly defined to solve the problem identified. Relates resources with aims.
- The next four aim to improve its execution:
  ** Evaluation of the difference,** which establishes whether the program actually implemented corresponds to the one that was planned, and whether the resources available correspond to the resources planned.
  **Evaluation of the structure (accreditation),** which clarifies whether the resources are suitable for achieving the desired results, and establishes the fit between resources and standards.
  **Analysis of productivity,** which examines whether optimal use has been made of the resources. Relates resources with processes.
  **Evaluation of the process (audit),** which examines whether the activities proposed or conducted are appropriate for achieving the desired results. Relates processes with standards.
- The last two steps identify the effects of the servuction:
  **Analysis of efficiency,** which assesses the relation between resources and results.
  **Analysis of efficacy and effectiveness, **which evaluates the results obtained with the servuction. Relates results with services.

## STRUCTURE OF THE ARTICLE

In this article we describe the stages involved in the design of a new health product and correlate them with the types of evaluation that should be applied at each point in the process. Our discussion will address general aspects of servuction, but will also focus on the design of a particular service, created to care for patients with severe COPD.

## STAGES OF THE PROJECT

1. Background - Previous Epidemiological Studies
2. Hypothesis
3. Aims
4. Product Design - Conceptualization of the New Model
5. Evaluation of the Suitability and Quality of the Servuction
6. Clinical Design for Output Evaluation
7. Implementation of the Model
8. Output Evaluation.
9. Conclusions.
10. Innovations Contributed by the Study

### 1 Background – Previous Epidemiological Studies (Strategic Evaluation)

#### 1.1 Prevalence of COPD.

The prevalence of chronic bronchitis (CB) is high, reaching 11.6% in our area of reference [9] and the prevalence of COPD was found to be 7.2% (Table **1**) [9]. As this is a progressive disease in a high percentage of cases, a not insignificant number of these patients need home oxygen therapy (HOT) in the final stage of their disease. This treatment has proved its ability to increase life expectancy in these patients [10].

#### 1.2 Health expenditure deriving from chronic diseases.

It is well known today that health expenditure rises as a person ages and as life expectancy increases. In the US, health expenditure deriving from the consumption of health services due to acute processes increases more slowly than expenditure due to long-term care [11]. Therefore, health expenditure is likely to increase both due to the increase in life expectancy and to the long-term care requirements of the elderly population [11].

#### 1.3 Current organizational model of health care in Catalonia.

There is no clear model for patients with severe COPD, a high percentage of whom present respiratory insufficiency. Currently, care for these patients may take three different forms: a) primary care, with occasional visits to the hospital pneumologist; b) a mix of primary and in-hospital care; c) in-hospital care by the pneumologist. Given the absence of gatekeeping at different levels in the system, the first two sets of patients effectively form a single group. In practice this means that these patients generate a high number of unnecessary or avoidable visits at hospital emergency services (HES) which does not seem likely to fall in the short term in spite of the adoption of preventive measures [12].

#### 1.4 Increase in the use of hospital emergency services.

The situation of HES care has been a matter of concern for many years for the health community and for society in general. All developed countries have witnessed an increase in HES use, rising in Spain from 9.2 million visits in 1984 to 15.3 million in 1994 [3]. To a large extent the increase can be attributed to a disproportionate rise in the number of patients making indiscriminate use of the HES and presenting with trivial complaints [3]. Patients tend to have more faith in the effectiveness of the HES services than of other areas in the health system such as primary care, which are often beset with serious organizational problems (see Table **2**).

#### 1.5 Comparison with alternative health models.

Countries in western Europe have made great efforts to design health care models that provide satisfactory clinical coverage at affordable cost. Numerous studies have compared the merits of home care, hospital at home, primary or hospital-based care, or mixed models such as short hospitalization with early hospital discharge).

Among these models, home hospitalization programs are often considered to represent a cost-effective alternative to conventional hospitalization [30]. However, few studies have explored the issue in depth and the results are occasionally contradictory [31]. Coast and cols [31] attribute these contradictions to the lack of reproducibility and comparability. Shepperd and cols published two studies in 1998 [32, 33] the first of which focused on the clinical viability of hospital at home and the second was a cost minimization study to assess its economic viability. COPD patients and patients convalescing from a hysterectomy were the only ones who expressed preference for conventional hospitalization. In the second article, on cost minimization, they noted that hospital at home for COPD patients was more expensive than conventional hospitalization [33]. They concluded that home care of patients with a relatively high degree of independence seems to be cost-effective, although patients who require a high level of nursing care should be attended by means of conventional hospitalization. However, we cannot deny that hospital at home may in the future constitute a useful complementary form of hospital care [34].

### 2 Hypothesis

The null hypothesis is that the design of a servuction for providing care for patients with COPD and respiratory insufficiency, using evidence-based medicine techniques, can’t: a) Improve patients’ clinical symptoms and quality of life. b) Reduce the costs of care for the financer and provider [35].

### 3 Aims

- To design a specific, novel servuction for patients with severe COPD using evidence-based medicine techniques.
- To evaluate the design of the servuction.
- To evaluate the repercussion of the new service for: a) effectiveness ; b) quality of life; c) efficiency: economic costs for the service supplier (the hospital) and for the financer (the Catalan Health Service, SCS).

### 4 Product Design – Conceptualization of the New Model

#### 4.1 Conceptualization

To design a new model of medical care for patients with chronic respiratory failure (CRF) following COPD, so as to reduce a) the number of unnecessary visits in primary care centres, other hospital departments and the emergency services at the CPT and b) the number of hospitalizations.

*In the design of the servuction, we follow the principles described by Eiglier [8] and apply the evidence-based medicine techniques supported by the Cochrane Collaboration.*

#### 4.2 Features of the Servuction (Program Analysis and Evaluation of the Differences)

##### 4.2.1 Clients.

To avoid service overload, the target population was defined as patients with CRF after COPD in our area of reference.

We defined clearly that the servuction was aimed at patients with chronic respiratory failure (CRF) only following COPD.

##### 4.2.2 Physical support.

Material support and environment.

###### 4.2.2.1 Material support.

Consumables required.

*The treatment required the use of a spirometer, a gasometer and a hemoxymeter from the functional assessment unit of the hospital’s pneumology service. By avoiding the doubling up of materials and spaces, we also develop economies of scale*.

###### 4.2.2.2 Environment.

The location of the service.

*Patients were seen in an office on the first floor of the Taulí building at the CPT, next to the respiratory function assessment lab. The office should be on the same floor as the assessment lab, to keep patient movement to a minimum - these elderly patients have considerable walking difficulties due to their dyspnea-.* 

##### 4.2.3 Contact personnel.

A junior physician and a nurse from the functional assessment unit.

*The program did not incur any extra expenditure for the CPT.*

#### 4.3 Capacity of the Servuction (Accreditation)

##### 4.3.1 Calculation of the number of potential beneficiaries.

The capacity of the new service, that is, the potential number of clients, should be calculated. This is a critical point. Just as in a clinical trial, the “sample size” should be estimated, even though in this case the aim is not to evaluate a variable of efficacy but to evaluate the minimum and maximum number of patients to attend. Obviously the figure will differ if we accept all patients with COPD or those with COPD and CRF.

*Using data from our own studies [9, 36] we determined that the prevalence of chronic home oxygen therapy (HOT) had fallen from 94 to 63 /100,000 inhabitants [36] between 1991 and 1995. This meant that there were 223 patients on the HOT program in our area, of whom 164 were followed at the hospital and the other 59 by a pneumologist at an outpatient service not attached to the our hospital. We then determined the number of frequently hospitalized patients (those admitted to hospital three times a year or more); the total was 92. Finally a group of patients who did not require HOT but who might do so in the short or medium term were also included as possible candidates. We estimated this figure to be around 50% of those included on the HOT program (111 in the entire reference area).*                    

*With these data, we calculated the maximum and minimum figures of patients at the service to be: a) Maximum: Patients on HOT in the entire area + patients likely to require HOT in the short or medium term + frequently hospitalized patients = 223 + 111 + 92 = 426; b) Minimum: Patients on HOT followed at the hospital + patients controlled at the hospital and likely to require HOT in the short or medium term (50% of 164) + frequently hospitalized patients = 164 + 82 + 92 = 338.*                    

*So our patient total was likely to range between 300 and 400. The final number of 289 patients indicates that the fit of capacity of the servuction was slightly lower than predicted.*                    

##### 4.3.2 Calculation of the number of scheduled appointments.

This section refers to routine scheduling.

Our service operated three days per week. We predicted an average of 4 appointments per patient per year, though obviously some patients would require more frequent attention. Assuming the figure of 300 patients, with one appointment every three months, the number of scheduled appointments was 1,200 a year; that is, 109 a month (1,200 appointments/11 months), 25 a week, or 7-8 appointments a day.

##### 4.3.3 Calculation of the number of non-scheduled appointments.

This section refers to emergency visits.

*This figure is very difficult to calculate. Nevertheless, we assumed that the mean would not be above one per day, bearing in mind that some consultations can be resolved satisfactorily by telephone and that decompensations are less common in the summer.*                    

##### 4.3.4 Total rate of appointments per day.

scheduled visits plus emergency consultations = 8-9 visits / day.

##### 4.3.5 Time management.

Time devoted to the service by the physician and nurse.

##### 4.3.6 Variability in seasonal demand.

It is well known that consultations due to decompensations in patients with COPD are more frequent in winter. Furthermore, the law requires [37] that the indication of HOT should be reviewed annually.

*To avoid overload at this service, we scheduled appointments for HOT and the renewal of authorization during the summer months, when fewer emergency consultations were expected.*                    

#### 4.4 Management of Patient Flows and Modification of Demand

Almost immediately, the management of patient flows should keep the waiting list within acceptable limits. Patients referred to this service were filtered by a “gatekeeper”, either a hospital pneumologist in the case of hospitalized patients or the head of the pneumology service who prioritized referrals from primary care.

*The time management plan proved to be accurate, as the initial provisions were able to cover the demand both for scheduled examinations and for emergency care. Seasonal demand has a major effect on the design of a product of this type. De la Iglesia and cols [38] established in a study of 232 patients that the number of admissions in winter was 36.2%, spring 28%, summer 12.9% and autumn 22.8%. So the initial decision for scheduling the standard visits for HOT in the summer months when there was less pressure from emergency admissions also proved sensible.*                

*In all, 3,589 consultations were made - some way below the predicted figure of around 4,800. It is difficult to estimate the number of emergency consultations because both in-hospital visits and telephone consultations are classed together here: normally, of course, patients do not have access to an emergency phone number.*                

#### 4.5 Internal Marketing

For a new product, having too few clients is as serious a problem as having too many. The last logistical problem we should mention is the possible lack of support (due mainly to a lack of information) from other hospital or primary care doctors who might refer patients to this new service.

*We acted at three different levels to raise our product’s profile. During a clinical session at the hospital we described the service to the rest of the medical staff who might refer patients. In the program of clinical sessions at primary care centres in our reference area we included COPD and informed participants of the launch of this service.*                

### 5 Evaluation of the Quality of the Servuction (Productivity Analysis)

As our evaluation criteria we decided to take the fit between the forecasts and the results for the number of visits (to assess the success of the planning of the new service) and a quality control of the new product. Post hoc analyses are always risky; the clinical and economic variables to evaluate must be clearly defined a priori.

*To measure the quality of the model we assessed its clinical effectiveness, its repercussion on patients’ quality of life and its efficiency (see below, paragraph 8).*

### 6 Clinical Design for Output Evaluation (Process Evaluation)

- The type of treatment should be clearly specified. The application of international regulations is always a guarantee of clarity.
  *The patients were treated according to a protocol based on the “Guidelines of the American Thoracic Society” [39] and the local government regulations [37] for the control of home oxygen therapy.*
- The type of study must be defined. Studies of effectiveness such as this one are often observational studies that evaluate cost minimization.
  *We performed an observational study with historical control*.
- Duration and follow-up time must be clearly stated, as in any scientific study.
  *The study lasted 5 years and the follow-up period for patients was 1 year.*

#### Population

Determination of sample size: in our assessment of indicators of effectiveness such as the number of emergency consultations and the number of hospitalisations, we calculated the sample of patients needed to draw conclusions.

*The experience of the research group, which observed a reduction similar to that recorded by other authors in the number of hospital admissions, was considered valid and reliable [40]. These data have been published elsewhere [35].*

Inclusion and exclusion criteria**: **these criteria have been defined elsewhere [35].

*The first important point in the design of this section is to establish the number of patients to evaluate. This is directly linked to the design of the study. We might have considered studying two groups of patients prospectively with two different types of intervention: treatment at our new specialized service compared with the standard situation in the Spanish National Health Service which owes more to chance than to a clearly established plan. This design has the advantage of being prospective in both groups but the real clinical reliability of the results might be difficult to extrapolate to other contexts since the control group may include many different types of medical care. In a recent publication Farrero and cols [41] studied 122 patients also followed up over a mean period of 1 year. Their study differed from ours in that the patients were treated in two groups with different levels of intervention.*

#### Methodology

Patient recruitment: subjects were recruited from all patients referred and admitted to our specialized service.

Program and description of instruments used: described elsewhere [35].

### 7 Implementation of the Model

*As specified in section 6, the model was implemented at the pneumology service of the CPT, which receives patients from both urban and rural environments*.

### 8 Evaluation of Results (Effectiveness and Efficiency Analysis)

The units of measurements were [35]:

- *Number of visits forecast and visits performed during the period*.
- *Clinical variables: spirometry, arterial gasometry, carboxyhemoglobin, nº of admissions to the ward, emergencies, and days of hospitalization*.
- *Economic variables, both for the financer (the Catalan Health Service, SCS) and for the provider (the hospital); mean cost of visits at hospital outpatient services, emergencies and hospitalisation, mean cost per patient, and aggregate costs*.
- *Assessment of quality of life using the Chronic Respiratory Disease Questionnaire*.

#### 8.1 Benefits obtained.

They are specified elsewhere [35].

Post hoc considerations.

In the literature there are no data on this type of health care model and so it is impossible to carry out reliable comparisons.

### 9 Conclusions

The conclusions should be based exclusively on the results of the product evaluated.

*In our case we obtained the following conclusions: a) the design of this specialized was satisfactory; b) management of patients by a hospital pneumologist at this service was more effective and efficient than mixed management by a GP and a hospital specialist (pneumologist or internist and slowed down the deterioration in their quality of life.*

#### Post hoc considerations

**Fig. (5).**
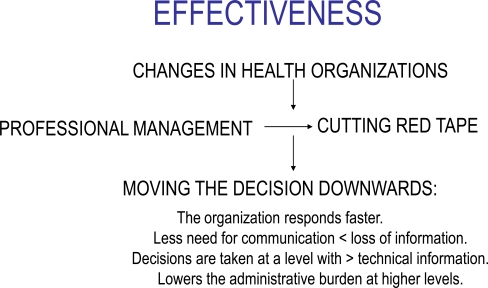


- *It is accepted today that many clinical decisions are the result of organizational habits or routines. Therefore, the more clearly defined the area of action, the more consistent the treatment that health professionals provide for specific pathologies; the more closely they comply with international guidelines, the lower the variance in their performance (thus reducing the variations in medical practice-VMP-). In addition, the further down the ladder the clinical decisions are taken, the faster the organization responds - which in medical terms tends to avoid neglected represent greater effectiveness (Fig. **5**) by avoiding neglences [42]*.
  *There is a notable geographical variation in the consumption of hospital resources both in Spain [43] and abroad [44]. Brook [45] and other authors attribute part at least of this variability of consumption of health services to organizational considerations. Our specialized service introduces notable improvements in this area*.

*Finally, since the criteria for referring patients to this specialized service were strictly medical, we believe that there is no risk of unfairness or the presence of influence costs, and the application of the “Eskimo economy” is also avoided [44]. The cost/opportunity ratio was also beneficial*.

### 10 Innovations Provided by the Model

In the final evaluation of a new health care model, the innovations it provides must be considered.

*Our design: a) complied with the recommendations of the Dawson report, the design of the study takes full account of the needs of the population, identified thoroughly in two earlier epidemiological studies performed between 1990 and 1995; b) the care program for COPD patients incorporates the methodology of evidence-based medicine for clinical management; c) the new model represents an important qualitative change, by limiting access to the system (introducing gatekeeping at various levels of the health care organization); d) A clinical and economic evaluation (effectiveness and efficiency) is presented of the improvements achieved by the new product; e)The behaviour of the new product is Paretian.*

## Figures and Tables

**Table 1.** Prevalence of COPD as a Function of Severity^1^

Degree of Bronchial Obstruction	Prevalence
FEV_1 _< 80%	7.2%
FEV_1_ < 75%	5.6%
FEV_1_ < 70%	3.6%
FEV_1_ < 65%	2.4%
FEV_1_ < 50%	1%
FEV_1_ < 35%	0.4%

**Table 2.** Percentage of Non-Urgent Cases Seen in Emergency Wards in Spain

Study	Year	% Non-Urgent Cases Seen in Emergency Wards
Castillo^13^	1986	58.6
Muiño^14^	1988	37
Balanzo^15^	1989	78.9
Diego^16^	1990	35
Ibañez^17^	1991	44.9
Rodriguez^18^	1992	65
Anton^19^	1992	65
Alonso^20^	1993	47.9
Cubero^21^	1994	60
Marco^22^	1994	55
Gonzalez-Grajera^23^	1995	49.5
Lapeña^24^	1996	69
Sansa^25^	1996	56-72
Llorente^26^	1996	24.1
Escobedo^27^	1997	54.7
Oterino^28^	1999	26.8
Sempere^29^	1999	29.6
